# Oral Cancer's New Enemy: *Goniothalamus umbrosus* Targets Oral Squamous Cell Carcinoma and Spare Human Gingival Fibroblast Cells

**DOI:** 10.1055/s-0044-1801278

**Published:** 2025-01-09

**Authors:** Nuraini Che Aziz, Basma Ezzat Mustafa Alahmad, Muhanad Ali Kashmoola, Widya Lestari, Nik Mohd Mazuan Nik Mohd Rosdy, Khairani Idah Mokhtar

**Affiliations:** 1 Department of Fundamental Dental Medical Science, Kulliyyah of Dentistry, International Islamic University Malaysia, Kuantan, Pahang, Malaysia; 2 Department of Dentistry, Bilad Alrafidain University College, Diyala, Iraq; 3 Faculty of Dentistry, Universiti Teknologi MARA, Jalan Hospital, Sungai Buloh, Selangor, Malaysia

**Keywords:** *Goniothalamus umbrosus*, oral squamous cell carcinoma, human gingival fibroblast, selective cytotoxicity

## Abstract

**Objective** Oral squamous cell carcinoma (OSCC) is the prevailing type of oral cancer, representing poor prognosis and elevated mortality rates. Major risk factors for OSCC include the use of tobacco products, alcohol consumption, betel quid chewing, and genetic mutation. *Goniothalamus umbrosus* is traditionally consumed by cancer patients to fight against tumor growth. To date, research on the anticancer potential of *G. umbrosus* in oral cancer remains deficient. This study aimed to evaluate the anticancer potential of *G. umbrosus* in OSCC cell lines (SCC-15 and HSC-3) and compare its cytotoxic activity on human gingival fibroblast (HGF) cell lines.

**Material and Methods** Leaves of *G. umbrosus* were cleaned, air dried, ground, and soaked for 24 hours with methanol and hexane repeatedly three times, respectively. Pooled extracts of each solvent were then dried with a rotary evaporator. Anticancer potential of *G. umbrosus* extracts was evaluated on two OSCC cell lines (SCC-15 and HSC-3) and a normal HGF cell line incubated for 24, 48, and 72 hours by 3-(4,5-dimethylthiazol-2-yl)-2,5-diphenyltetrazolium bromide (MTT) assay. The cytotoxicity of cisplatin was assessed as a positive control. Morphological changes of cells were observed under an inverted microscope.

**Results** MTT assay revealed that *G. umbrosus* methanol extract (GUME) displayed moderate anticancer activity on SCC-15, HSC-3, and HGF cell lines with IC _50_ values of 126.67, 90.5, and 87.33 µg/mL following 72 hours' incubation times, respectively. *G. umbrosus* hexane extract (GUHE) exerted moderate anticancer activity against SCC-15 and HSC-3 cell lines with IC _50_ values of 171 and 174 µg/mL, respectively, but weak cytotoxicity against the HGF cell line with IC _50_ value of 343.5 µg/mL. Cisplatin exerted a strong cytotoxic impact on both OSCC and HGF cell lines. Morphological observation revealed the characteristics of cells undergoing apoptosis.

**Conclusion** The findings show that GUHE was more selective in inhibiting the proliferation of oral cancer cells than GUME by exerting moderate cytotoxicity on OSCC cell lines and weak cytotoxicity in HGF cells, while GUME exerted moderate cytotoxicity on both. These findings suggest a more targeted anticancer effect by GUHE as compared with cisplatin, which exerted nonselective cytotoxic activity. These findings provide a groundwork for the development of more targeted plant-based treatment for oral cancer.

## Introduction

Cancer of the oral cavity represents the 16th most common neoplasm globally with 389,846 new cases documented in 2020. 1 In addition, 188,438 deaths were reported making oral cancer the 15th in the global mortality ranking due to cancer. 1 In Malaysia, oral cancer is ranked 18th among the most commonly diagnosed cancer with 826 new cases and 440 death cases reported in 2022. 2 While it is not the most commonly diagnosed cancer, it is still considered a life-threatening disease as the function of mastication, deglutition, and speech will be distorted.

Oral squamous cell carcinoma (OSCC) is the predominant histological subtype of oral cancer, which accounts for more than 90% of cases. 3 It can develop in any location of the mucosa, but the tongue and floor of the mouth are the most frequently affected area. 4 The usual presentation of oral cancer is an ulcerated lesion, with a central necrotic area surrounded by raised borders. 5 The treatment strategies in oral cancer therapy include surgery, radiotherapy, and chemotherapy. However, despite the massive progress in diagnosis and treatments, the 5-year survival rate of oral cancer patients is still below 50%. 6 In addition, chemotherapeutic drugs also possess unbearable side effects, which affect patient's survival and quality of life. Hence, many patients tend to choose alternative therapy, which is the traditional medicine as it is thought to be a safer option for treating oral cancer. Ideal anticancer agents should have minimal side effects while effectively killing cancer cells.

Drugs derived from natural origin are considered ideal candidates for anticancer drug development since most anticancer leads are derived from plants. Examples include vincristine (Vinca alkaloid from *Catharanthus rosea* G. Don.) and paclitaxel ( *Taxus brevifolia* Nutt.). 7 Furthermore, herbal medicine has been widely consumed by many traditional medicine practitioners for general well-being and a majority of them demonstrate anticancer effects by either inhibiting the progression of cancer through the prevention of deoxyribonucleic acid (DNA) synthesis, which leads to malignancy or by impeding the proliferation of premalignant cells with DNA damage. 8 However, the information about traditional medicine is still deficient due to a lack of scientific approach among traditional practitioners and the knowledge on medicinal plants is usually transferred by verbal communication from one generation to another, which might lead to miscommunication in transferring knowledge.

*Goniothalamus umbrosus* is a shrub with smooth, thin, fibrous strong aromatic bark and upright blackish cylindrical trunk that can grow up to 3 m in height. It is traditionally consumed for general well-being by grinding the leaves, encapsulated and prepared as a tonic drink for the management of abortion, postpartum health care, and fever. 9 It has been reported to exert an anticancer effect against breast cancer cell lines (MCF-7) and cervical cancer cells (HeLa). 10 11 12 So far, the cytotoxic effect of this plant against normal cells has only been reported on Vero cells, showing weak cytotoxicity, which might indicate that this plant exerts a different cytotoxicity effect against normal cells. 13 In general, the cytotoxic effect of *G. umbrosus* has mainly been evaluated on cancer cell lines with limited studies comparing the effect on its normal counterpart. The current study aims to evaluate the cytotoxic activity of *G. umbrosus* in OSCC cell lines and compare its impact on normal human gingival fibroblast (HGF) cell lines. New fundamental knowledge regarding the cytotoxic effect induced by *G. umbrosus* on oral cancer cells and its protective effect on HGF will serve as a foundation for considering the potential use of this plant as a prospective anticancer agent.

## Materials and Methods

### Preparation of Methanol and Hexane Extract of *G. umbrosus*

The leaves of *G. umbrosus* were obtained from Machang, Kelantan, Malaysia. The fresh leaves were cleaned from any debris and dried at room temperature (25°C). The weight of dried leaves was recorded and ground into powder. The powdered leaves of *G. umbrosus* were subjected to maceration in methanol and hexane solvents. An amount of 800 g of powdered *G. umbrosus* was macerated in 1,500 mL of methanol (methanol:water, 80:20 v/v) for 24 hours at room temperature (25°C), followed by filtration with Whatman No. 1 filter paper. 12 The process was repeated another three times to ensure exhaustive extraction. 14 The filtrates were pooled and solvent was dried using a rotary evaporator (50°C, 150 mbar). On the other hand, 90 g of powdered *G. umbrosus* was macerated in 1,500 mL of hexane under the same condition. 10 The filtrates were pooled and concentrated using a rotary evaporator (50°C, 200 mbar). The weight of the dried concentrated crude extract was measured to calculate the percentage yield. The percentage yield was calculated using the formula: (weight of crude extract (g)/weight of dried plant material (g)) × 100. 15 The dried extract was stored in a refrigerator (4°C), until further analysis.

### Preparation of Stock Solution

An amount of 10 mg of dried *G. umbrosus* methanol extract (GUME) and hexane extract (GUHE) was dissolved in 1 mL of methanol, respectively, and sonicated for approximately 3 to 5 minutes until the extract was completely dissolved. It was then diluted with complete media to a 1-mg/mL concentration for downstream analysis.

### Cell Culture

SCC-15 (CRL-1623) and HGF (PCS-201–018) cell lines were purchased from the American Type Culture Collection (ATCC, Manassas, Virginia, United States). HSC-3 was generously given by Dr. Wastuti Hidayati Suriyah from PLANETIIUM, International Islamic University Malaysia. SCC-15 cell line was cultured in Dulbecco's Modified Eagle Medium (DMEM)/F12 medium supplemented with 10% fetal bovine serum (FBS), 100 U/mL of penicillin/streptomycin, and 400 ng/mL of hydrocortisone (Sigma-Aldrich, United States). HSC-3 and HGF cell lines were cultured in DMEM media supplemented with 10% FBS and 100 U/mL of penicillin/streptomycin. All cell lines were cultured in 25 cm ^2^ flasks at 37°C in a relative humidity of 95% and 5% CO _2_ . The medium was replaced every 48 hours and cultured until cells reached 80 to 90% confluency, which was monitored daily using an inverted microscope.

### 3-(4,5-Dimethylthiazol-2-yl)-2,5-Diphenyltetrazolium Bromide Assay

The cytotoxic activity of GUME and GUHE were evaluated using a 3-(4,5-dimethylthiazol-2-yl)-2,5-diphenyltetrazolium bromide (MTT) assay. SCC-15 cell line was seeded in 96-well plates at a density of 5 × 10 ^4^ cells/100 µL/well while HSC-3 and HGF cell lines were seeded at a density of 2 × 10 ^4^ cells/100 µL/well. These cell densities were predetermined in the preliminary study. After 24 hours of seeding, the media was replaced with fresh media containing various concentrations of GUME and GUHE ranging from 1,000, 500, 250, 125, 62.5, 31.25, 15.62, and 7.81 µg/mL according to a previous study 12 with slight modification. Treated cells were further incubated for 24, 48, and 72 hours. The untreated cells served as a negative control. Cells were also treated with cisplatin as a positive control 16 following 72-hour incubation time. At the end of the respective incubation time, 10 µL of MTT solution was introduced to each well and further incubated for 3 hours at 37°C. Subsequently, the MTT reagent was discarded and dimethyl sulfoxide was added to dissolve the formazan crystal formed by reduction of the MTT reagent. Cell viability was determined by measuring optical density (OD) at 570 nm with a microplate reader. The OD of the control sample was obtained from untreated cells. Each experiment was performed in quadruplicate and independently repeated thrice. The values obtained were then used to plot the percentage of cell viability against concentrations of extracts used in the experiment. The IC _50_ values (50% inhibitory concentration) were determined from the curve.

### Morphological Observation

The morphological changes in the cells particularly related to apoptosis such as cell shrinkage, elongation and attachment were observed and photographed at 100× magnification using an inverted microscope. These characteristics were compared with untreated control cells which maintained normal morphology and adhered to the flask.

## Results

### Percentage Yield

The methanol extract of *G. umbrosus* yielded 28%, whereas the hexane extract exhibited a yield of 3.61%.

### Cytotoxic Effect

**Fig. 1:**
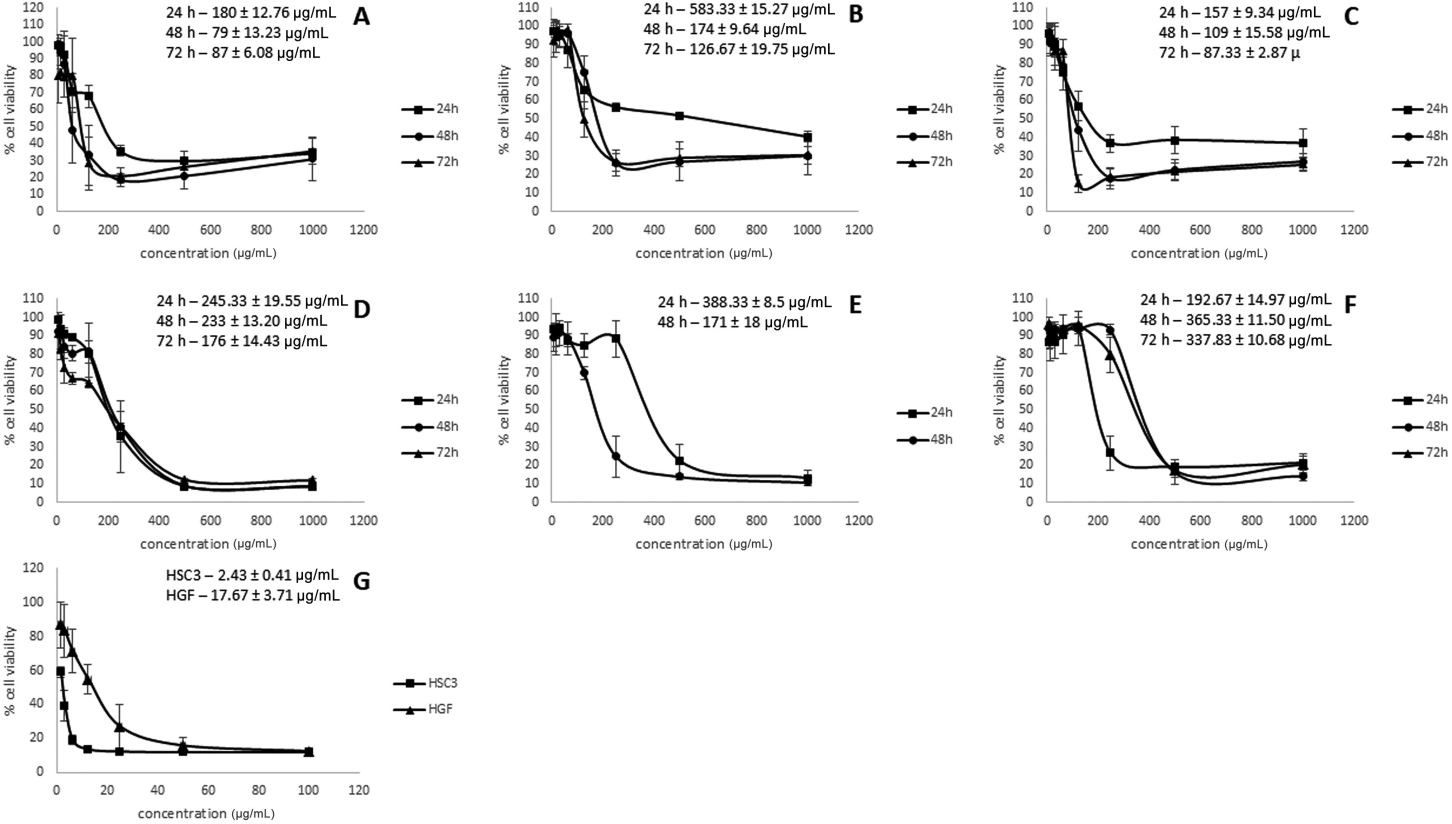
Cytotoxic activity of *G. umbrosus* methanol extract on HSC-3 cell line ( **A** ), SCC-15 cell line ( **B** ), and human gingival fibroblast (HGF) cell line ( **C** ). Hexane extract of *G. umbrosus* on HSC-3 cell line ( **D** ), SCC-15 cell line and HGF cell line ( **F** ), and cisplatin on HSC-3 and HGF cell line ( **G** ).

The cytotoxic effect of GUME and GUHE against SCC-15, HSC-3, and HGF cell lines was evaluated using the MTT assay. GUME decreases SCC-15 and HSC-3 cell viability in a concentration- and time-dependent manner. It exerted moderate cytotoxicity against HSC-3 with IC _50_ values of 180, 79, and 87 µg/mL following 24-, 48-, and 72-hour incubation with extracts, respectively ( Fig. 1A ). GUME demonstrated weak cytotoxicity against SCC-15 following 24-hour incubation time with IC _50_ value of 583 µg/mL and moderate cytotoxicity with IC _50_ values of 174 and 126.67 µg/mL following 48- and 72-hour incubation times, respectively, as depicted in Fig. 1B . GUME also depicts moderate cytotoxicity against HGF cell lines in concentration- and time-dependent manner by displaying IC _50_ values of 157, 109, and 87.33 µg/mL following 24-, 48-, and 72-hour incubation times, respectively, as represented in Fig. 1C . GUHE inhibited the proliferation of SCC-15 and HSC-3 and HGF cell lines in a concentration- and time-dependent manner. Fig. 1D demonstrated that GUHE inhibited the growth of the HSC-3 cell line with IC _50_ values of 245.33, 233, and 176 µg/mL following 24-, 48-, and 72-hour incubation times, respectively. GUHE also exerted moderate cytotoxic activity against the SCC-15 cell line with IC _50_ values of 388.33 and 171 µg/mL following 24- and 48-hour incubation times ( Fig. 1E ). GUHE demonstrated moderate cytotoxicity against the HGF cell line in all incubation times with IC _50_ values of 192.67, 365.33, and 337.835 µg/mL ( Fig. 1F ).

Cisplatin displayed strong cytotoxic activity against HSC-3 with IC _50_ values of 2.43 and 17.67 µg/mL in the HGF cell line ( Fig. 1G ) following 72-hour incubation time.

### Morphological Changes

**Fig. 2:**
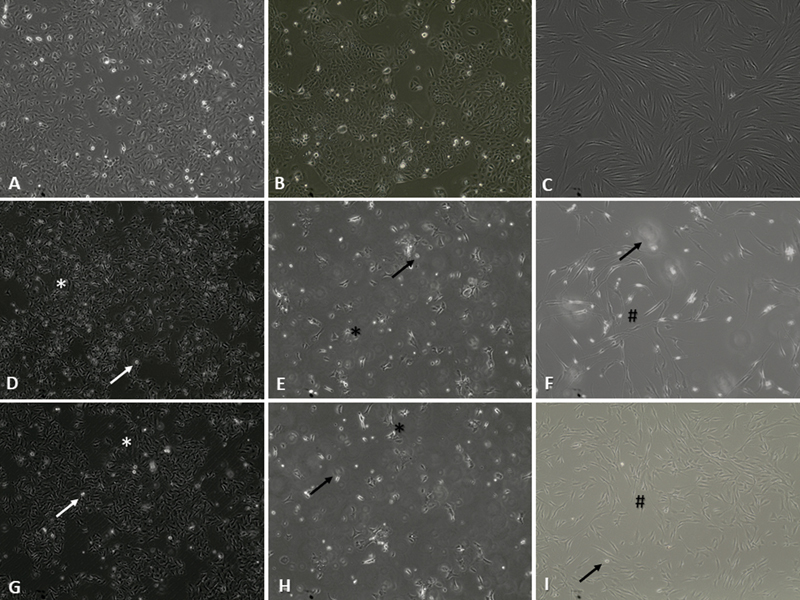
Morphology of untreated cells HSC-3 ( **A** ), SCC-15 ( **B** ), and human gingival fibroblast (HGF) ( **C** ). Morphological changes in cells treated with methanol extract of *G. umbrosus* in HSC-3 ( **D** ), SCC-15 ( **E** ), and HGF ( **F** ). Morphological changes in cells treated with hexane extract of *G. umbrosus* in HSC-3 ( **G** ), SCC-15 ( **H** ), and HGF ( **I** ). Magnification: 100×. * cell shrinkage, # cell elongation, floating cells.

**Fig. 3:**
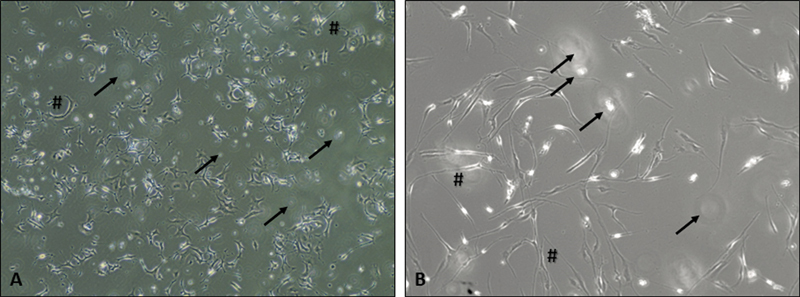
Morphology of HSC-3 ( **A** ) and human gingival fibroblast (HGF) ( **B** ) cells treated with cisplatin. Magnification: 100×. * cell shrinkage, # cell elongation, floating cells.

The morphological changes of HSC-3, SCC-15, and HGF cells treated with GUME and GUHE were examined under an inverted microscope. The typical epithelial characteristic of untreated HSC-3 ( Fig. 2A ) changed into a noticeable reduction in cell number with the presence of floating cells, cell shrinkage, and cell elongation after incubation with GUME ( Fig. 2D ) and GUHE ( Fig. 2E ). The morphology of untreated SCC-15 ( Fig. 2B ) was also changed to similar features observed in HSC-3 following treatment with GUME ( Fig. 2E ) and GUHE ( Fig. 2H ). The morphology of untreated HGF cells ( Fig. 2C ) was observed to be more elongated with a visible decrease in cell number following treatment with GUME ( Fig. 2F ) compared with untreated cells. Meanwhile, the morphology of HGF cells treated with GUHE ( Fig. 2I ) was similar to untreated cells with only a few floating cells observed. In addition, the morphology of HSC-3 treated with cisplatin ( Fig. 3A ) was altered to more shrinking and elongated with a significant number of floating cells. A similar pattern of morphological changes was also observed in HGF cells treated with cisplatin ( Fig. 2B ).

## Discussion

The current study aims to investigate the cytotoxic effect of *G. umbrosus* extract on two OSCC cell lines, which are HSC-3 and SCC-15. Both cell lines were originally excised from the human tongue with HSC-3 displaying more aggressive behavior as it was harvested from a metastatic lymph node of tongue squamous cell carcinoma. 17 Meanwhile, SCC-15 is a primary OSCC of the tongue. 18 The cytotoxic effect of *G. umbrosus* was also assessed on HGF cells to compare its effect on normal cells and determine the selectivity of the extract in inhibiting the growth of OSCC cell lines without being detrimental to surrounding normal cells.

The present study evaluated the cytotoxicity of two extracts of *G. umbrosus* , methanol and hexane. Methanol and hexane were chosen as solvents to extract *G. umbrosus* due to their opposite polarities, which allows the extraction of a wide range of compounds. Methanol is a polar solvent that is effective in extracting polar compounds including phenolics and flavonoids, which are often associated with the bioactivity of the plant extract. 19 In contrast, hexane is a polar solvent that is ideal for extracting nonpolar compounds such as lipids and terpenoids. 19 These solvents were chosen to enhance the extraction efficacy ensuring a wide spectrum of compounds are included for the analysis. The higher yield with methanol (28%) suggests that *G. umbrosus* is rich in polar bioactive compounds. On the other hand, the lower yield of hexane (3.61%) is consistent with its selectivity in extracting only nonpolar compounds, which are usually present in minute quantities compared with polar compounds. This finding was in line with prior studies of extracting *Bauhinia variegata* 20 and *Populus* Salicaceae bark 19 where both studies produced more methanol yield compared with hexane.

The cytotoxic effect of extracts was evaluated by obtaining the IC _50_ values from the dose–response curve following the MTT assay. The IC _50_ value represents the concentration of extract required to inhibit 50% of the cell viability, therefore lower IC _50_ values suggest greater extract potency against cell lines tested. Plant extracts that demonstrated IC _50_ values between 20 and 200 µg/mL are considered to exert moderate cytotoxicity and IC _50_ values exceeding 200 µg/mL indicate low cytotoxicity against cell line tested. 21 The GUME exerted moderate cytotoxic activity in a time- and concentration-dependent manner against all cell lines tested. This indicates that GUME was toxic to both cancer and normal cell lines and did not have a selective inhibitory effect. This finding was consistent with a previous report that showed that the methanol extract of *G. umbrosus* exerts moderate cytotoxic activity on HeLa cells with an IC _50_ value of 48 µg/mL. 12 Meanwhile, GUHE exerted moderate cytotoxicity against both SCC-15 and HSC-3 cell lines and low cytotoxicity against the HGF cell line. This indicates that GUHE was more selective in inhibiting the proliferation of cancer cells yet not detrimental to normal cells. This finding was in line with a previous report that demonstrated the hexane extract of *G. umbrosus* has a potent cytotoxic effect against breast cancer cell line (MCF-7) with an IC _50_ value of 20 µg/mL. 10 The cytotoxicity of cisplatin was tested as a positive control and it was found to possess strong cytotoxicity against both HSC-3 and HGF cell lines with an IC _50_ value of < 20 µg/mL, which suggests that cisplatin does not employ a selective inhibitory effect. This finding was consistent with a previous finding that it exerted a nonselective inhibitory effect in SCC-15 and HGF cell lines after exposure to cisplatin followed by irradiation with cold atmosphere plasma. 22 Platinum-based drug cisplatin is a widely used drug in the chemotherapeutic regimen for oral cancer patients. However, cisplatin lacks selectivity whereby it inhibits the growth of highly proliferating cells, which include cancer cells and normal healthy fast growing cells such as hair and nail cells leading to the development of many unbearable side effects of chemotherapy for instance alopecia, brittle nails, nausea, and so forth. 23 While the cytotoxicity of GUHE against cancer cells is incomparable with cisplatin, it nonetheless exhibited a selective inhibitory effect compared with cisplatin, which may offer a therapeutic advantage by minimizing the side effects on normal cells. These findings highlight the potential of GUHE for further analysis as an adjuvant or alternative treatment in oral cancer therapy.

The hallmark of apoptosis including cell shrinkage, cell elongation, and the presence of floating cells 24 were observed in this study ( Fig. 2 ). In addition, more floating cells and reduced cell number were observed in the HGF cells treated with GUME compared with GUHE, which aligns with the MTT assay findings. These findings suggest that the anticancer effect of both GUME and GUHE on SCC-15 and HSC-3 cell lines occurs through apoptosis with GUME also triggering cell death in HGF cells. This finding was in line with previous studies that demonstrated that the hexane extract of *G. umbrosus* induces apoptosis in breast cancer cells (MCF-7) by the appearance of morphological changes such as membrane blebs, DNA condensation, and fragmentation observed under an inverted microscope. 10 In addition, the methanol extract of *G. umbrosus* leaves was also found to have an antiproliferative effect with morphological changes such as cell shrinkage, cell elongation, cell rounding, and vacuolization in cervical cancer cells (HeLa). 12 Other studies revealed that the ethyl acetate extract of *G. umbrosus* also triggered apoptosis by the appearance of membrane bleb and reduced number of live cells in breast cancer cell line (MCF-7). 11 Furthermore, the morphology of HSC-3 and HGF cells was noticeably altered following treatment with cisplatin, which supports the MTT assay finding that cisplatin exhibited nonselective cytotoxicity against HSC-3 and HGF cell lines. These observations were consistent with a previous study where the morphology of OSCC and HGF cell lines was altered following exposure to cisplatin. 22

Apoptosis is a fundamental mechanism for keeping a balance between cell proliferation and cell death. Activation of certain enzymes, in particular, the caspases and other molecules will determine the death or survival of cells. 24 Morphological characteristics of apoptosis include chromatin condensation, nuclear fragmentation, cell rounding, and cellular volume reduction (pyknosis), which lead to the alteration of cytoplasmic organelles and loss of membrane integrity in the final stage. 24 These morphological changes result from biological perturbations that occur via caspase activation, DNA and protein degradation, membrane alteration, and recognition of apoptotic bodies by phagocytic cells. 25

One limitation of this study is the lack of recent literature on the anticancer effect as the most relevant study was dated back in 2009 10 11 and only one study was done in 2018. 12 The lack of recent studies is probably due to the difficulty in obtaining the resource as this plant was only found in certain places in Malaysia. 9 Additionally, due to technical issues in the laboratory, the study on the cytotoxic effect on SCC-15 could not be done for 72 hours even though the results for 24 and 48 hours were promising.

Treatment with both GUME and GUHE has the potential to induce apoptosis in SCC-15 and HSC-3 cell lines as observed through the cytotoxic and morphological changes. Future research should be conducted to isolate and characterize the bioactive compound in *G. umbrosus* as well as investigate the precise molecular pathways, particularly in gene and protein expression related to apoptosis to further elucidate the underlying mechanism of *G. umbrosus* .

## Conclusion

The current study on the cytotoxicity effect of GUME and GUHE suggests that both extracts exerted moderate cytotoxicity against OSCC cell lines tested. Interestingly, GUHE showed a better promising anticancer effect in exerting a more targeted cytotoxicity impact by selectively inhibiting the proliferation of the OSCC cell lines without harming the HGF cell line as compared with GUME and cisplatin that showed nonselective cytotoxicity against cancer and normal cell lines. These findings highlight the need for further analysis to elucidate the molecular mechanism that contributes to the apoptosis characteristic observed in this study. This result provides a theoretical reference for the development of more targeted plant-based adjuvant therapy for oral cancer.
